# Timing of pharmacologic venous thromboembolism prophylaxis initiation for trauma patients with nonoperatively managed blunt abdominal solid organ injury: a systematic review and meta-analysis

**DOI:** 10.1186/s13017-022-00423-1

**Published:** 2022-04-25

**Authors:** Tyler Lamb, Tori Lenet, Amin Zahrai, Joseph R. Shaw, Ryan McLarty, Risa Shorr, Grégoire Le Gal, Peter Glen

**Affiliations:** 1 Division of General Surgery, Department of Surgery, The Ottawa Hospital, 725 Parkdale Avenue, Ottawa, ON K1Y 1J8 Canada; 2 School of Epidemiology and Public Health, Faculty of Medicine, University of Ottawa, Ottawa, ON Canada; 3 Division of Hematology, Department of Medicine, The Ottawa Hospital, Ottawa, ON Canada; 4 The Ottawa Hospital Research Institute, Ottawa, ON Canada; 5 Division of Urology, Department of Surgery, The Ottawa Hospital, Ottawa, ON Canada; 6 Library and Information Sciences, The Ottawa Hospital, Ottawa, ON Canada; 7 Ottawa Regional Trauma Program, The Ottawa Hospital, Ottawa, ON Canada

**Keywords:** Blunt trauma, Thrombosis, Hepatic injury, Splenic injury, Nonoperative management

## Abstract

**Background:**

Blunt abdominal solid organ injury is common and is often managed nonoperatively. Clinicians must balance risk of both hemorrhage and thrombosis. The optimal timing of pharmacologic venous thromboembolism prophylaxis (VTEp) initiation in this population is unclear. The objective was to evaluate early (< 48 h) compared to late initiation of VTEp in adult trauma patients with blunt abdominal solid organ injury managed nonoperatively.

**Methods:**

Embase, MEDLINE, and the Cochrane Central Register of Controlled Trials were searched from inception to March 2021. Studies comparing timeframes of VTEp initiation were considered. The primary outcome was failure of nonoperative management (NOM) after VTEp initiation. Secondary outcomes included risk of transfusion, other bleeding complications, risk of deep vein thrombosis (DVT) and pulmonary embolism, and mortality.

**Results:**

Ten cohort studies met inclusion criteria, with a total of 4642 patients. Meta-analysis revealed a statistically significant increase in the risk of failure of NOM among patients receiving early VTEp (OR 1.76, 95% CI 1.01–3.05, *p* = 0.05). There was no significant difference in risk of transfusion. Odds of DVT were significantly lower in the early group (OR 0.36, 95% CI 0.22–0.59, *p* < 0.0001). There was no difference in mortality (OR 1.50, 95% CI 0.82–2.75, *p* = 0.19). All studies were at serious risk of bias due to confounding.

**Conclusions:**

Initiation of VTEp earlier than 48 h following hospitalization is associated with an increased risk of failure of NOM but a decreased risk of DVT. Absolute failure rates of NOM are low. Initiation of VTEp at 48 h may balance the risks of bleeding and VTE.

**Supplementary Information:**

The online version contains supplementary material available at 10.1186/s13017-022-00423-1.

## Background

Blunt trauma is the most common mechanism of traumatic injury, accounting for approximately 80% of trauma-related hospital admissions [1]. Nonoperative management (NOM) is the standard of care for hemodynamically stable patients with blunt abdominal solid organ injury. This includes close monitoring, serial abdominal examinations, and early interventional procedures such as angioembolization [2, 3].

Following initial trauma-induced coagulopathy, patients transition to a hypercoagulable state within 24 to 48 h [4]. Trauma patients are at high risk of venous thromboembolic (VTE) events with rates of deep vein thrombosis (DVT) as high as 60% in some populations [5]. Failure of NOM due to bleeding and development of VTE are divergent considerations. Pharmacologic prophylaxis has been shown to effectively reduce VTE complication rates among patients with traumatic injuries [6]. When deciding on the timing of pharmacologic VTE prophylaxis (VTEp) initiation, clinicians must balance the risk of hemorrhage from injuries with the risk of VTE.

Guidelines support the early initiation of pharmacologic VTEp with unfractionated heparin (UFH) or low molecular weight heparin (LMWH) in patients with blunt abdominal solid organ injury managed nonoperatively [7–10]. However, the optimal timing of VTEp initiation has remained unclear. Reported rates of failure of NOM due to bleeding are as high as 11–20% for patients with liver and spleen injuries, with most failures occurring within 48 h of injury [2, 11]. Recently, a systematic review and meta-analysis including ten studies published prior to April 2020 concluded that VTEp could be safely initiated within 48 h [12]. However, a 2021 study including 3223 patients with isolated blunt abdominal solid organ injuries conversely found a significantly increased risk of bleeding in those who received VTEp at earlier than 48 h [13]. We therefore conducted a systematic review of the literature to determine the optimal timing for initiation of pharmacologic VTEp among patients with blunt abdominal solid organ injury managed nonoperatively, considering the most contemporary evidence.

### Objectives

The aim of this systematic review was to determine the risks and benefits of early (< 48 h) compared to late (≥ 48 h) initiation of pharmacologic VTEp in adult trauma patients with blunt abdominal solid organ injury managed nonoperatively. The primary objective was to determine whether early initiation of pharmacologic VTEp is associated with a difference in the rate of failure of NOM, compared to late initiation. Secondary objectives were to evaluate differences in risk of DVT and pulmonary embolism (PE), blood transfusion, mortality, and other bleeding complications.

## Methods

The Cochrane Handbook for Systematic Reviews of Interventions and The Preferred Reporting Items for Systematic Review and Meta-Analysis (PRISMA) checklist guided protocol development and reporting [14, 15]. The protocol was registered with PROSPERO (CRD42021241143).

### Eligibility criteria

The population of interest included adult patients with blunt abdominal solid organ injury managed nonoperatively. Studies including adolescent patients (age ≥ 13) were also included. Randomized controlled trials and observational cohort studies were eligible for inclusion. Case reports, case series with < 5 patients, and case–control studies were excluded. Conference abstracts and grey literature were eligible for inclusion.

Studies were included irrespective of study-defined timing of VTEp initiation. We categorized patients based on a priori definitions of early (< 48 h from admission to hospital) and late (≥ 48 h) initiation. To be eligible, studies had to report results according to the timing of VTEp initiation. Only studies assessing LMWH or UFH at conventional prophylactic doses were eligible. Inclusion was restricted to English and French languages. There were no date restrictions. If duplicate patient populations from large databases were encountered in multiple studies, only the largest and most recent study was included.

### Information sources and search strategy

The search strategy is included in Additional file 1: Appendix A. Indexed databases incorporated in the search included Embase, MEDLINE, and the Cochrane Central Register of Controlled Trials. Reference lists of relevant systematic reviews were reviewed to identify eligible studies not captured by our database search.

Titles, abstracts, and potentially eligible full texts were reviewed independently and in duplicate. Eligibility criteria for full-text screening were applied using an electronic eligibility checklist (Additional file 2: Appendix B). Data collection was performed independently and in duplicate. All discrepancies were resolved by consensus or a third author.

### Outcomes

#### Primary outcome

The primary outcome was the proportion of patients in each study arm who failed NOM and required either surgical or radiological intervention for hemostasis after initiation of pharmacologic VTEp. Patients who were initially treated with angioembolization prior to VTEp initiation were not considered to have failed NOM (Additional file 3, Additional file 4).

#### Secondary outcomes

Secondary outcomes included need for packed red blood cell (pRBC) transfusion and the number of units of pRBCs transfused following initiation of VTEp, risk of VTE (DVT and PE), mortality, and other bleeding complications (e.g., intracranial hemorrhage).

### Risk of bias in individual studies

Risk of bias for each included study was assessed in duplicate by independent reviewers using the Risk of Bias in Non-Randomized Studies of Interventions (ROBINS-I) tool [16].

### Data synthesis

Random-effects models were used for meta-analyses, as they provide more conservative estimates than the fixed-effects model, allowing for greater heterogeneity between studies. Statistical heterogeneity was examined using the *I*^2^ statistic, with categories of low (0–30%), moderate (31–60%), and substantial (61–100%) [14]. A homogenous treatment effect was assumed when different prophylactic LMWH/UFH formulations or doses were evaluated within the same study. The GRADE approach was used to rate the quality of evidence for estimates derived from meta-analyses [17].

## Results

### Study selection

**Fig. 1:**
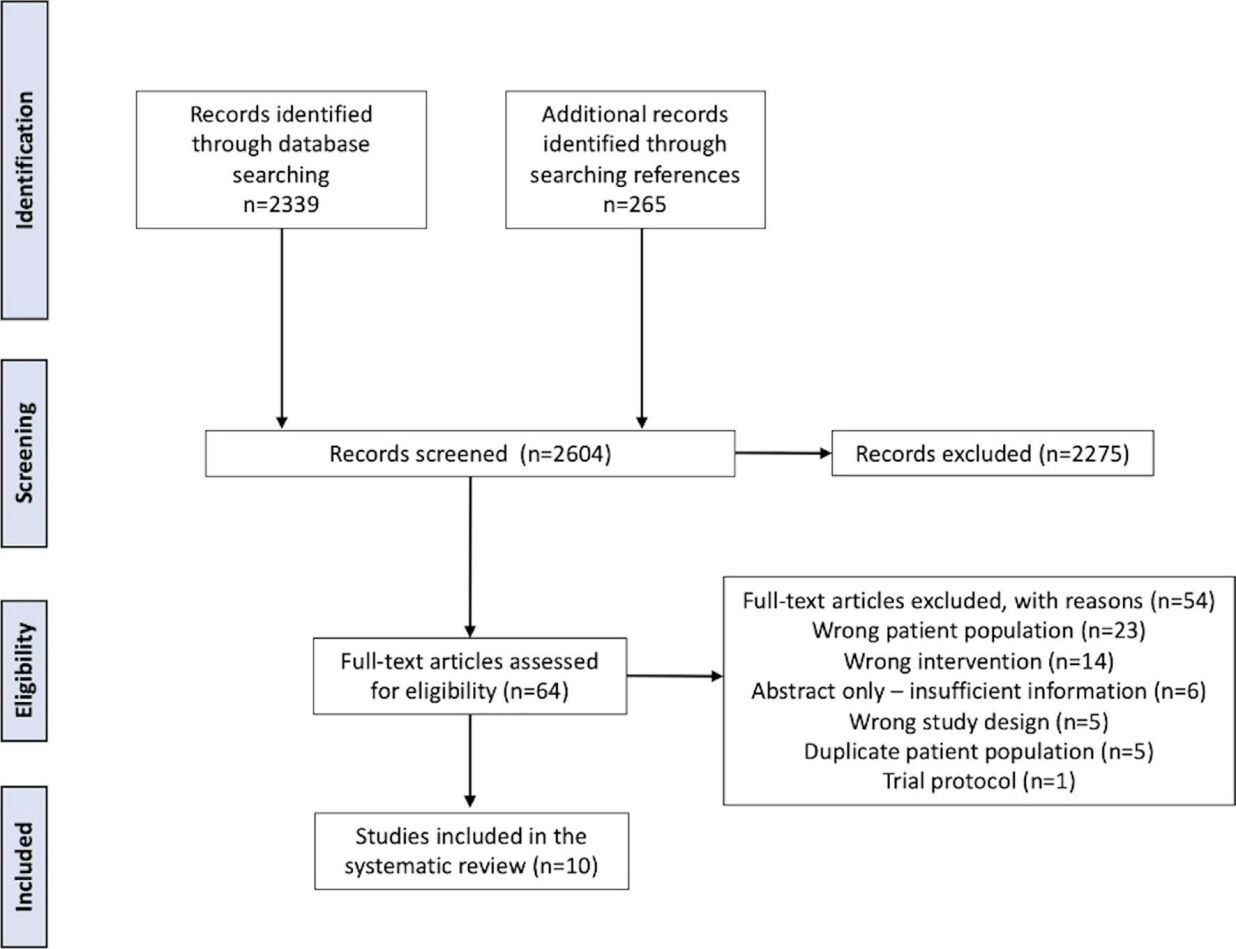
PRISMA flow diagram

Our literature search identified 2339 records through database searching and an additional 265 records through searching references, for a total of 2604 records. Title and abstract screening excluded 2275 records and 64 full-text studies were assessed. Ten studies including 4642 patients met full eligibility criteria (Fig. 1) [1, 13, 18–25]. Reasons for exclusion included wrong patient population (*n* = 23), wrong intervention (*n* = 14), wrong study design (*n* = 5), abstract with insufficient information for inclusion (*n* = 6), and a trial protocol without results (*n* = 1). A further five studies using the Trauma Quality Improvement Program (TQIP) database were excluded due to duplicate patient populations. No additional studies were identified from references of included texts or relevant systematic reviews.

### Study characteristics

Study characteristics are presented in Table 1. One study using the TQIP database contributed 69% of patients to the included cohort (3223/4642) [13]. No interventional or randomized trials were identified. Nine retrospective cohort studies and one prospective cohort study were included [25]. Most studies included patients with any abdominal solid organ injury, although three studies restricted their population to patients with specific organ injuries (*n* = 2 for splenic injuries [18, 22], *n* = 1 for hepatic injuries) [19]. LMWH or UFH, or a combination thereof, was included as VTEp in all studies, with dosing based on standardized time intervals rather than weight in seven studies [1, 18, 20–24]. Details on dosing regimens were unavailable for three studies [13, 19, 25].

**Table 1:** Baseline study characteristics

Study ID	Intervention groups	Year	Study design	Study center design	Patient population	Number of patients (*n*)	Adjustment for confounding	ISS *Mean(SD)* *Median [IQR]*	Age *Mean (SD)* *Median [IQR]*	Concurrent Head Injury *n (%)*	Hospital LOS (days)	ICU LOS (days)
Alejandro et al. *(N* = *114)*	≤ 48 h	2003	Retrospective cohort	Single center	Splenic injuries	50	None	18.5 (9.1)	36.7 (19.1)	–	12.5 (16.6)	11.2 (8.2)
	> 48 h					64		16.8 (8.3)	38.3 (16.0)		10.8 (13.1)	14.7 (9.7)
Datta et al. *(N* = *72)*	≤ 48 h	2009	Retrospective cohort	Multicenter	Hepatic injuries	27	None	22 (mean)	37 (mean)	0/27 (0)	14	8
	> 48 h					45		25 (mean)	42 (mean)	0/45 (0)	26	9
Eberle et al. *(N* = *111)*	< 72 h	2011	Retrospective cohort	Single center	Blunt SOI	41	Multivariable Regression	21.9 (9.7)	37.5 (15.7)	9/41 (22.0)	–	–
	≥ 72 h					70		24.6 (10.0)	36.3 (14.3)	27/70 (40.0)	–	–
Joseph et al. *(N* = *116)*	≤ 48 h	2015	Retrospective cohort	Single center	Blunt SOI	58	Propensity Score Matching	17 [12–19]	39.5 (18.2)	–	3.9 (2.9)	2.1 (1.9)
	48–72 h					29		17 [14–24]	44.3 (21.4)		4.1 (3.6)	2.5 (2.1)
	≥ 72 h					29		17 [13–26]	45.1 (22.9)		4.8 (4.1)	2.4 (2.3)
Rostas et al. *(N* = *328)*	< 48 h	2015	Retrospective cohort	Multicenter	Blunt SOI	103	None	18.7 (mean)	–	3/103 (2.9)	–	–
	48–72 h					54		22.6 (mean)		7/54 (13.0)	–	–
	> 72 h					171		36.9 (mean)		12/171 (7.0)	–	–
Kwok et al. *(N* = *256)*	< 24 h	2016	Retrospective cohort	Single center	Splenic injuries	23	None	19 [14–29]	40 (17)	0/23 (0)	13 (15.0)	5 (11.0)
	24–48 h					91				0/91 (0)	8 (6.0)	2 (5.0)
	48–72 h					65				0/65 (0)	14 (15.0)	7 (13.0)
	> 72 h					77				0/77 (0)	17 (17.0)	8 (14.0)
Murphy et al. *(N* = *162)*	< 48 h	2016	Retrospective cohort	Single center	Blunt SOI	78	None	21 (9)	43 (19)	0/78 (0)	7 [4–9]	3 [1–6]
	≥ 48 h					84		17 (9)	41 (18)	0/84 (0)	4 [3–7]	4 [3–7]
Khatsilouskaya et al. *(N* = *142)*	≤ 72 h	2017	Retrospective cohort	Single center	Blunt SOI	80	None	17 [11]	38.4 [29.4]	10/80 (12.5)	–	–
	> 72 h					62		29 [21]	36.8 [30.7]	20/62 (32.3)	–	–
Schellenberg et al. *(N* = *118)*	≤ 48 h	2019	Prospective cohort	Single center	Blunt SOI	61	None	17 [14–22]	36 [27–54]	5/61 (8.0)	6 [4–11]	3 [2–6]
	> 48 h					57		22 [17–27]	36 [27–56]	18/57 (32.0)	14 [7–35]	7 [4–12]
Gaitanidis et al. *(N* = *3223)*	< 48 h	2021	Retrospective cohort	Multicenter	Blunt SOI	1832	Multivariable Regression	14 [10–17]	34 [24–50]	0/1832 (0)	–	–
	48–72 h					703		16 [13–20]	33 [24–52]	0/703 (0)	–	–
	> 72 h					688		16 [13–21]	37 [25–55]	0/688 (0)	–	–

*SOI* solid organ injury, *ISS* injury severity score, *LOS* length of stay

Injury severity score (ISS) was similar between studies, although three studies limited their patient population to those with higher ISS [19, 23, 24]. ISS was generally higher in groups with later VTEp initiation. Three studies excluded patients with significant head injuries [13, 19, 22]. The proportion of patients with head injuries was variably reported, but varied considerably. Other confounding variables, including orthopedic injuries, use of tranexamic acid (TXA), protocolized bed rest, and use of mechanical VTEp, were seldom reported. American Association for the Surgery of Trauma (AAST) grading, stratified by organ type and VTEp timing, is presented in Additional file 5: Table S1. In general, AAST grade was higher in the delayed groups, with few high-grade injuries in the early VTEp groups. Follow-up in all studies was limited to hospital discharge. Routine screening for asymptomatic VTE was not performed in any study.

### Risk of bias

**Fig. 2:**
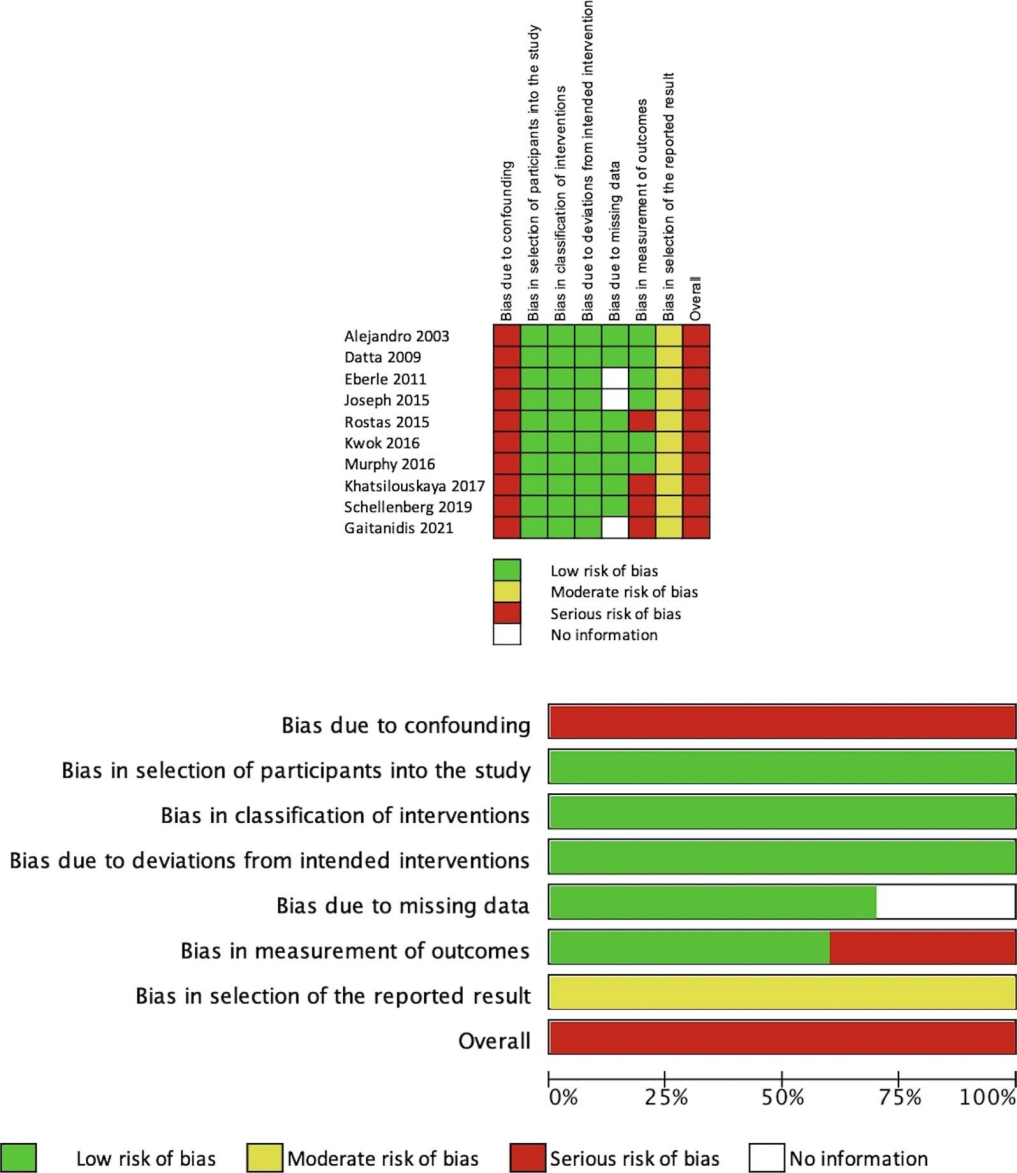
Risk of bias assessment using the ROBINS-I tool

The risk of bias assessment is presented in Fig. 2. All studies were considered to be at serious risk of bias, primarily due to concerns for residual confounding despite attempts at adjustment using either propensity score matching or multivariable regression analysis. There were important differences between those receiving early and delayed VTEp; notably, patients in the delayed groups tended to have higher AAST grades and ISS.

### Study outcomes

Outcome data are reported in Table 2. Meta-analysis of adjusted data was not possible due to a lack of studies reporting adjusted data, methodological differences between adjustment techniques, and variable reporting of treatment effects (e.g., one study reported adjusted *p* values without the associated adjusted OR). Subgroup analyses were not possible due to insufficient reporting of data stratified by prespecified subgroups. All studies were judged to be at serious risk of bias, precluding a sensitivity analysis of studies at low risk of bias.

**Table 2:**
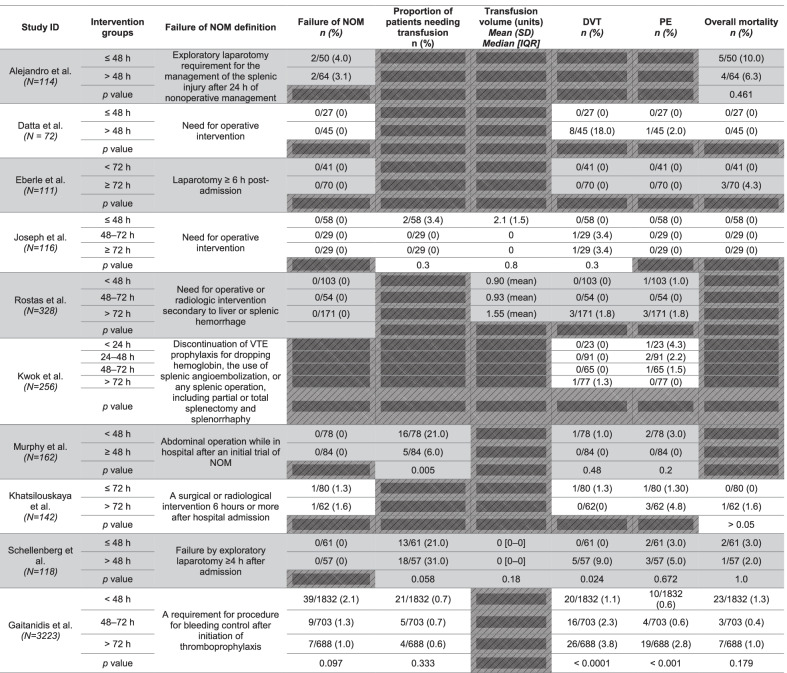
Summary of outcome data for included studies

*NOM* nonoperative management, *DVT* deep vein thrombosis, *PE* pulmonary embolism

### Primary outcome

#### Failure of NOM

**Fig. 3:**
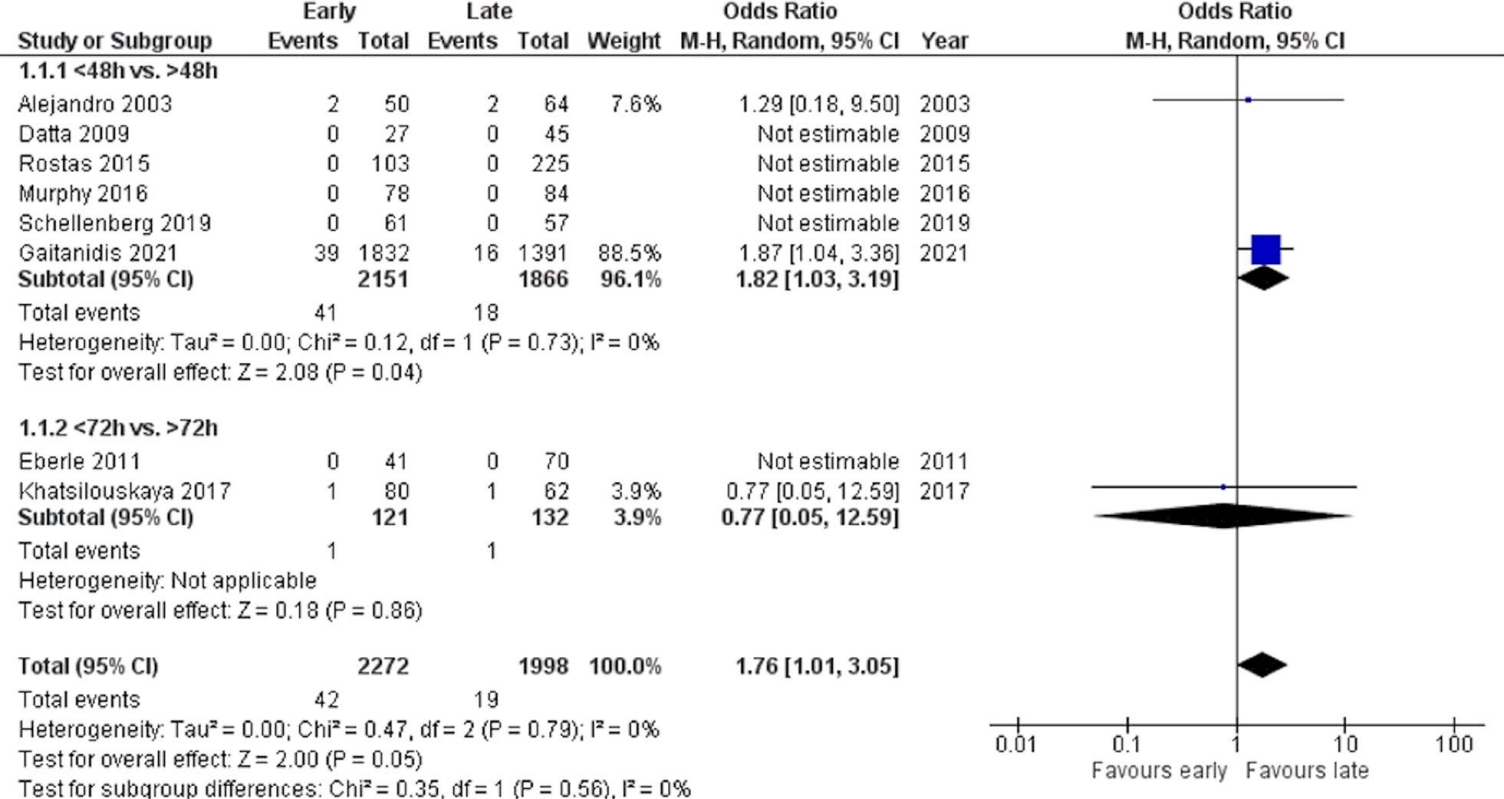
Failure of NOM after VTE prophylaxis initiation (unadjusted data)

Meta-analysis of unadjusted data from three studies [13, 18, 24] showed a statistically significant increase in the rate of failure of NOM with early VTEp initiation (OR 1.76, 95% CI 1.01–3.05, *p* = 0.05) (Fig. 3). Statistical heterogeneity was low (*I*^2^ = 0%). Failure rates of NOM after VTEp initiation were reported by all but one study [22] and were generally low, with most studies reporting no NOM failures in any group. Among all patients who failed NOM, 49 (80.3%) patients required surgical intervention, while 12 (19.7%) were managed with angioembolization.

### Secondary outcomes

#### Transfusion requirement

Risk of transfusion following VTEp initiation was reported by four studies [1, 13, 23, 25]. Meta-analysis demonstrated no significant difference in risk of transfusion between the early and late groups (OR 1.56, 95% CI 0.55–4.48, *p* = 0.41) (Additional file 6: Fig. S1). Statistical heterogeneity was high (*I*^2^ = 77%). Transfusion volume requirements following VTEp were reported by three studies, with all reporting a mean requirement of less than two units [1, 21, 25]. Meta-analysis of transfusion volume data was not possible due to the number of reporting studies and unreported measures of dispersion.

#### Bleeding complications

Two studies reported risk of bleeding complications. Rostas et al. reported no bleeding complications in either group [21], while Gaitanidis et al. reported higher rates of “either receiving blood product transfusions after initiation of thromboprophylaxis or undergoing intervention for bleeding control (surgery or angioembolization)” in the early group [13].

#### Risk of VTE

**Fig. 4:**
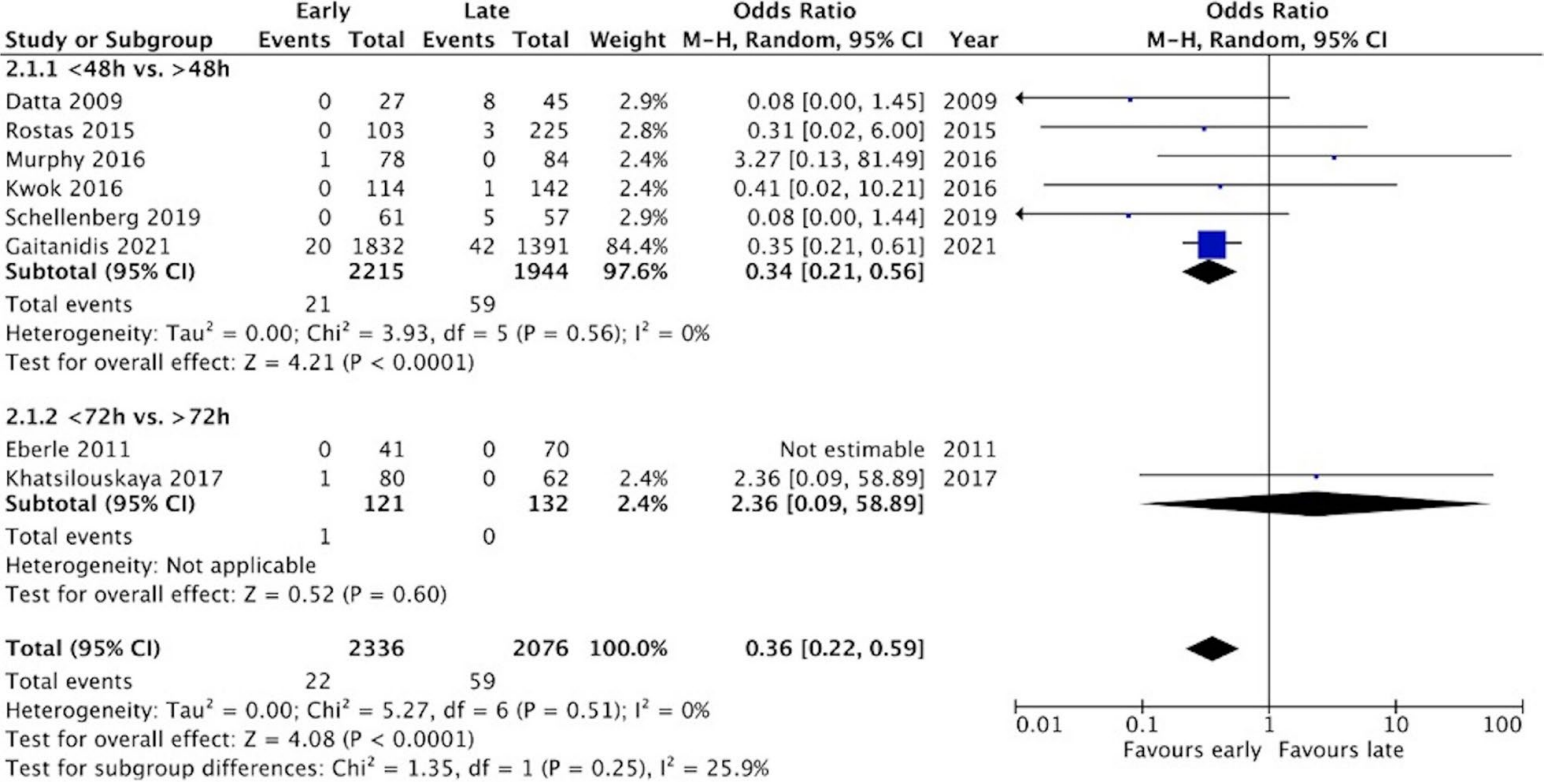
Risk of DVT (unadjusted data)

Risk of VTE was reported by all but one study [18]. Two studies reported a significantly greater risk of DVT in the delayed VTEp group [13, 25], while one study reported a significantly greater risk of PE [13]. Meta-analysis revealed a statistically significant lower risk of DVT in the early group (OR 0.36, 95% CI 0.22–0.59, *p* < 0.0001) (Fig. 4). Statistical heterogeneity was low (*I*^2^ = 0%). The risk of PE was also lower in the early group, but did not reach statistical significance (OR 0.58, 95% CI 0.27–1.25, *p* = 0.16) (Additional file 7: Fig. S2). Statistical heterogeneity was low (*I*^2^ = 17%).

#### Mortality

Mortality was reported by seven studies [1, 13, 18–20, 24, 25]. No study reported a statistically significant difference in mortality. Meta-analysis revealed no statistically significant difference between the early and delayed groups (OR 1.50, 95% CI 0.82–2.75, *p* = 0.19) (Additional file 8: Fig. S3). Statistical heterogeneity was low (*I*^2^ = 0%).

### Reporting biases and certainty of evidence

Publication bias and small study bias could not be assessed given that all meta-analyses included fewer than ten studies [14]. A summary of the certainty of evidence is presented in Table 3. Outcomes including failure of NOM, transfusion requirements, PE, and mortality demonstrated a low or very low level of certainty using the GRADE framework [17]. There was moderate certainty in the reported reduction in DVT with early compared to late VTEp initiation.

**Table 3:** GRADE assessment for quality of evidence

Early VTE prophylaxis compared to late VTE prophylaxis for trauma patients with nonoperatively managed blunt abdominal solid organ injury					
Population: Trauma patients with nonoperatively managed blunt abdominal solid organ injury Intervention: Early (< 48 h) VTE prophylaxis Comparison: Late (≥ 48 h) VTE prophylaxis					
Outcomes	Anticipated absolute effects* (95% CI)		Relative effect (95% CI)	Number of participants (studies)	Certainty of evidence (GRADE)
	Risk with late VTE prophylaxis	Risk with early VTE prophylaxis			
Failure of NOM	10 per 1000	17 per 1000 (10 to 28)	OR 1.76 (1.01 to 3.05)	4270 (8 observational studies)	Low^a,b^
Transfusion Risk	21 per 1000	32 per 1000 (12 to 87)	OR 1.56 (0.55 to 4.48)	3503 (3 observational studies)	Very low^a,c,d^
DVT	28 per 1000	10 per 1000 (6 to 17)	OR 0.36 (0.22 to 0.59)	4412 (8 observational studies)	Moderate^a^
PE	16 per 1000	10 per 1000 (4 to 20)	OR 0.58 (0.27 to 1.25)	4412 (8 observational studies)	Low^a,d^
Mortality	11 per 1000	17 per 1000 (9 to 30)	OR 1.50 (0.82 to 2.75)	3780 (6 observational studies)	Very low^a,d,e^

GRADE Working Group Grades of Evidence

High Certainty: We are very confident that the true effect lies close to that of the estimate of the effect

Moderate Certainty: We are moderately confident in the effect estimate. The true effect is likely to be close to the estimate of the effect, but there is a possibility that it is substantially different

Low Certainty: Our confidence in the effect estimate is limited. The true effect may be substantially different from the estimate of the effect

Very Low Certainty: We have very little confidence in the effect estimate. The true effect is likely to be substantially different from the estimate of effect

*VTE* venous thromboembolism, *CI* confidence interval, *OR* odds ratio, *NOM* nonoperative management, *DVT* deep vein thrombosis, *PE* pulmonary embolism

*The risk in the intervention group (and its 95% CI) is based on the assumed risk in the comparison group and the relative effect of the intervention (and its 95% CI)

^a^Bias due to confounding and bias based on selection of the reported result

^b^Confidence interval is somewhat wide and does not cross the null effect but includes appreciable risk/benefit (OR 1.25) in the confidence interval. Also, the number of events is small

^c^Some overlap in confidence intervals across studies, but there is statistically significant, considerable heterogeneity. Also, there is some difference in the OR across studies

^d^Confidence interval is wide as it includes OR = 1 and an appreciable risk/benefit (OR 0.75 and 1.25). Also, the number of events is small

^e^Large *I*^2^ and variation in effect sizes is large

## Discussion

This systematic review identified ten observational studies including 4642 patients and evaluated the optimal timing for initiation of VTEp among trauma patients with blunt abdominal solid organ injury undergoing NOM. Failure of NOM was an infrequent outcome, but meta-analysis revealed that early VTEp initiation was associated with a statistically significant increase in the rate of failure of NOM. Additionally, there was a statistically significant lower risk of DVT in the early group. No significant differences were observed in risk of mortality or transfusion. Initiation of VTEp at 48 h may be ideal among patients with blunt solid organ injury undergoing NOM.

The uncertainty surrounding the optimal time to initiate VTEp reflects the difficulty of managing patients at high risk of both bleeding and thrombotic complications. The Western Trauma Association suggests starting VTEp 24 h post-injury [7], whereas the AAST recommends starting VTEp within 48 h of injury [10]. These recommendations are largely based on expert opinion. Studies identified in this review varied with respect to timing of initiation, with early initiation of prophylaxis most frequently defined as < 48 h [1, 13, 18, 19, 21, 23, 25]. Thromboelastographic studies have identified a transition from trauma-induced coagulopathy to a hypercoagulable state at 48 h among patients with blunt solid organ injury, suggesting that 48 h might provide the optimal balance of minimizing risk of both bleeding and VTE [26].

Most studies did not report any failures of NOM following initiation of VTEp in either study arm. The largest study in our review (using the TQIP database) included over 3000 patients and identified increased rates of failure of NOM and bleeding complications among patients with initiation of VTEp at < 48 h compared to at > 72 h (2.1% vs. 1.0%) [13]. Conversely, among other studies evaluating a cutoff of < 48 h for early initiation, 377 patients received VTEp within 48 h and only two patients experienced failure of NOM (0.5%) [1, 18, 19, 21, 23, 25]. The discrepant findings between Gaitanidis et al. and other included studies could be due to several factors. It was by far the largest included study in our review due to its use of a multinational database and had the greatest statistical power to detect a significant impact from earlier VTEp initiation on a rare outcome. With the exception of Gaitanidis et al., included studies were mainly single-center retrospective cohort studies reviewing local databases. The TQIP database includes over 850 community and academic trauma centers from across the USA and Canada, and there is likely to be substantial heterogeneity with respect to local practice patterns among reporting centers.

Importantly, the finding that early VTEp initiation was associated with a statistically significant increase in the rate of failure of NOM differs from the conclusions of a recent systematic review and meta-analysis [12]. As noted above, the findings were largely driven by the Gaitanidis et al.’s study, which was published in January 2021 and therefore could not be included in the review by Murphy et al. [12, 13]. It is also important to note that the Gaitanidis et al.’s study excluded patients with an extra-abdominal Abbreviated Injury Scale score greater than 3, thereby eliminating an important confounder that can impact the decision to start VTEp [13].

We did not identify a statistically significant difference in transfusion risk between early and late initiation of VTEp. However, the reported risk of transfusion was substantially higher among single-center studies compared to the multinational database study (e.g., 21–31% in the Schellenberg et al.’s study compared to 0.6–1.2% in Gaitanidis et al.) [13, 25]. These findings raise the possibility of underreporting of transfusion events in the TQIP database, as it would be expected that most patients who failed NOM due to bleeding would require transfusions as well.

The increased rate of VTE complications with delayed VTEp initiation observed among several studies highlights the importance of initiating VTEp as soon as is safely possible. No study performed routine VTE screening, so identified events were likely to be symptomatic and clinically relevant. Interpretation of these results is challenging due to variable follow-up across studies and study arms. The longer length of stay observed in the delayed group in several studies likely contributed to a greater risk for VTE and outcome identification. Our pooled unadjusted analyses were consistent with the results observed among the individual studies. PE was a less common outcome, and so the lack of statistical significance may be due to fewer events and lack of power to detect a difference.

Compared to the recent review by Murphy et al., our findings were similar with respect to VTE and transfusion requirements, but discordant for failure of NOM. Our review includes the most recent study in this area, as well as a 2009 study not included by Murphy et al. [13, 19] Additionally, our review differs in that we excluded a large study by Skarupa et al., as the more recent study by Gaitanidis et al. also utilized the TQIP database over a longer time period and therefore had some sample duplication, but limited their population to those without significant extra-abdominal injuries, which was a strength compared to the Skarupa et al.’s study [13, 27].

This review has several limitations. Importantly, all included studies were observational and prone to substantial bias. Patients in the delayed VTEp arm of each study had higher ISS, higher AAST injury grades, and longer hospital and intensive care unit admissions (Table 1, Additional file 5: Table S1). These findings may be due to surgeons appropriately deferring VTEp among severely injured patients perceived to be at increased risk of bleeding complications. This may therefore underestimate the impact of early VTEp initiation on the risk of failure of NOM. Despite several studies attempting to control confounding, given the nature of the intervention and observational nature of the data, it becomes impossible to eliminate or substantially mitigate this bias, as treating physicians were making a conscious decision on whether to delay VTEp. Additionally, some studies included patients with head injuries, which are a key factor in deciding on timing of initiation of VTEp. Furthermore, longer hospitalizations were noted in the delayed VTEp arm in several studies (Table 1). Accordingly, there is a significant risk of ascertainment bias, particularly for those outcomes occurring later during hospitalization (i.e., VTE). Lastly, we were unable to conduct a meta-analysis of adjusted data as most studies did not perform adjustment for confounders.

## Conclusions

The optimal timing for initiation of VTEp is a matter of ongoing debate and substantial interest in the trauma surgery community. Initiation at < 48 h appears to be the most frequently used definition for early initiation of VTEp among patients with blunt solid organ injury. Initiation of VTEp at 48 h among patients with low-grade injuries may balance the risk of bleeding complications and mitigate the risk of VTE associated with later prophylaxis initiation. Prospective research with careful control of confounding is needed to further evaluate the safety of this threshold. Moreover, standardizing follow-up duration, definitions of NOM, and outcomes of interest would facilitate future research and enable improved synthesis of results.

## Supplementary Information

**Additional file 1: Appendix A.** Search strategy.

**Additional file 2: Appendix B.** Additional study materials.

**Additional file 3: Appendix C.** Excluded full text citations and reasons.

**Additional file 4: Appendix D.** PRISMA checklist.

**Additional file 5: Table S1.** AAST Grading of injuries in included studies.

**Additional file 6: Fig. S1.** Risk of transfusion after initiation of VTE prophylaxis (unadjusted data).

**Additional file 7: Fig. S2.** Risk of PE (unadjusted data).

**Additional file 8: Fig. S3.** Risk of mortality (unadjusted data).

## Data Availability

The datasets generated and analyzed for this study are not presently publicly available but will be made available from the corresponding author on reasonable request.

## Abbreviations

NOM: Nonoperative management
VTE: Venous thromboembolism
DVT: Deep vein thrombosis
VTEp: Pharmacologic VTE prophylaxis
UFH: Unfractionated heparin
LMWH: Low molecular weight heparin
PE: Pulmonary embolism
PRISMA: Preferred Reporting Items for Systematic Review and Meta-Analysis
pRBC: Packed red blood cell
ROBINS-I: Risk of Bias in Non-Randomized Studies of Interventions
TQIP: Trauma Quality Improvement Program
ISS: Injury severity score
TXA: Tranexamic acid
AAST: American Association for the Surgery of Trauma
OR: Odds ratio
CI: Confidence interval
