# Supplementary material for: Timing of pharmacologic venous thromboembolism prophylaxis initiation for trauma patients with nonoperatively managed blunt abdominal solid organ injury: a systematic review and meta-analysis
Source: World J Emerg Surg. 2022 Apr 25;17:19. doi: 10.1186/s13017-022-00423-1 (PMC9036793; doi:10.1186/s13017-022-00423-1)
Supplement: Supplementary file 1 — Additional file 1: Appendix A. Search strategy. [file 13017_2022_423_MOESM1_ESM.docx]

**Appendix A.** Search strategy.

Database: Embase Classic+Embase <1947 to 2021 March 01> , Ovid MEDLINE(R) ALL <1946 to  March 01, 2021>, EBM Reviews - Cochrane Central Register of Controlled Trials <March 2021>

Search Strategy:

--------------------------------------------------------------------------------

1 Venous Thromboembolism/ and (pc.fs. or prevent*.tw,kf. or prophyla*.tw,kf. or chemoprophyla*.tw,kf.) (25152)

2 thromboprophyla*.tw,kf. (15085)

3 exp Anticoagulants/ and (pc.fs. or prevent*.tw,kf. or prophyla*.tw,kf. or chemoprophyla*.tw,kf.) (211134)

4 ((anticoagul* or anti coagul*) and (prevent* or prophyla* or chemoprophyla*)).tw,kf. (63181)

5 thromb* prophyla*.tw,kf. (8409)

6 ((Venous Thrombo* or vein thrombo* or vte or dvt) adj5 (prevent* or prophyla* or chemoprophyla*)).tw,kf. (32843)

7 (Dalteparin or Enoxaparin or Nadroparin or Tinzaparin or heparin* or lmwh*).tw,kf. and (pc.fs. or prevent*.tw,kf. or prophyla*.tw,kf. or chemoprophyla*.tw,kf.) (60321)

8 or/1-7 (258673)

9 "Wounds and Injuries"/ or Wounds, Nonpenetrating/ (290087)

10 (trauma or traumas).ti,kf. (191891)

11 exp Abdominal Injuries/ (203805)

12 ((abdom* or liver or spleen or splenic or kidney or renal or hepatic or solid organ*) adj2 (injur* or trauma* or ruptur* or bleed*)).tw,kf. (271443)

13 ((abdom* or liver or spleen or splenic or kidney or renal or hepatic or solid organ*) and (injur* or trauma* or ruptur* or bleed*)).ti. (148400)

14 or/9-13 (846643)

15 8 and 14 (7114)

16 exp animals/ not humans/ (19074457)

17 15 not 16 (4290)

18 17 use medall (1333)  Medline

19 exp venous thromboembolism/pc [Prevention] (22910)

20 thrombosis prevention/ (12732)

21 exp venous thromboembolism/ and (prevention/ or prophylaxis/ or post exposure prophylaxis/ or pre-exposure prophylaxis/ or chemoprophylaxis/) (11359)

22 *anticoagulation/ and (pc.fs. or prevent*.tw. or prophyla*.tw. or chemoprophyla*.tw.) (21515)

23 ((anticoagul* or anti coagul* or heparin or lmwh or Enoxaparin or Dalteparin or Tinzaparin or Nadroparin) and (prevent* or prophyla* or chemoprophyla*)).tw. (91712)

24 thromboprophyla*.tw. (14928)

25 thromb* prophyla*.tw. (8346)

26 ((Venous Thrombo* or vein thrombo* or vte or dvt) adj5 (prevent* or prophyla* or chemoprophyla*)).tw. (32756)

27 or/19-26 (140300)

28 exp *abdominal injury/ (90443)

29 abdominal injury/ or abdominal blunt trauma/ or abdominal contusion/ or abdominal organ rupture/ or digestive system injury/ or spleen injury/ (43782)

30 *injury/ (136968)

31 (trauma or traumas).ti. (176814)

32 ((abdom* or liver or spleen or splenic or kidney or renal or hepatic or solid organ*) adj2 (injur* or trauma* or ruptur* or bleed*)).tw. (268629)

33 ((abdom* or liver or spleen or splenic or kidney or renal or hepatic or solid organ*) and (injur* or trauma* or ruptur* or bleed*)).ti. (148400)

34 28 or 29 or 30 or 31 or 32 or 33 (617046)

35 27 and 34 (3030)

36 (exp animal/ or nonhuman/) not exp human/ (12170719)

37 35 not 36 (2828)

38 37 use emczd (1734)  Embase

39 Venous Thromboembolism/ and (pc.fs. or prevent*.tw,kw. or prophyla*.tw,kw. or chemoprophyla*.tw,kw.) (25233)

40 thromboprophyla*.tw,kw. (15427)

41 exp Anticoagulants/ and (pc.fs. or prevent*.tw,kw. or prophyla*.tw,kw. or chemoprophyla*.tw,kw.) (211953)

42 ((anticoagul* or anti coagul*) and (prevent* or prophyla* or chemoprophyla*)).tw,kw. (65156)

43 thromb* prophyla*.tw,kw. (8581)

44 ((Venous Thrombo* or vein thrombo* or vte or dvt) adj5 (prevent* or prophyla* or chemoprophyla*)).tw,kw. (33331)

45 (Dalteparin or Enoxaparin or Nadroparin or Tinzaparin or heparin* or lmwh*).tw,kw. and (pc.fs. or prevent*.tw,kw. or prophyla*.tw,kw. or chemoprophyla*.tw,kw.) (61101)

46 39 or 40 or 41 or 42 or 43 or 44 or 45 (260416)

47 "Wounds and Injuries"/ or Wounds, Nonpenetrating/ (290087)

48 (trauma or traumas).ti. (176814)

49 exp Abdominal Injuries/ (203805)

50 ((abdom* or liver or spleen or splenic or kidney or renal or hepatic or solid organ*) adj2 (injur* or trauma* or ruptur* or bleed*)).tw,kw. (273758)

51 ((abdom* or liver or spleen or splenic or kidney or renal or hepatic or solid organ*) and (injur* or trauma* or ruptur* or bleed*)).ti. (148400)

52 or/47-51 (835359)

53 46 and 52 (7119)

54 53 use cctr (246)  Cochrane

55 18 or 38 or 54 (3313)  Total

56 remove duplicates from 55 (2420)  Total after duplicates

57 56 use medall (1327)  Medline

58 56 use emczd (970)  Embase

59 56 use cctr (123)  Cochrane
