# Supplementary material for: Timing of pharmacologic venous thromboembolism prophylaxis initiation for trauma patients with nonoperatively managed blunt abdominal solid organ injury: a systematic review and meta-analysis
Source: World J Emerg Surg. 2022 Apr 25;17:19. doi: 10.1186/s13017-022-00423-1 (PMC9036793; doi:10.1186/s13017-022-00423-1)
Supplement: Supplementary file 2 — Additional file 2: Appendix B. Additional study materials. [file 13017_2022_423_MOESM2_ESM.docx]

**Appendix B.** Additional study materials.

Full Text Eligibility Form

| **Team Member Completing Form** | | | |  | | |
| --- | --- | --- | --- | --- | --- | --- |
| **Study ID** *(First author, year of publication)* | | | |  | | |
| **Study Characteristics** | **Eligibility Criteria** | | | | **Eligibility Criteria Met?**  *Yes/No/Unclear* | **Location in Text**  *(i.e. page/fig/table/other)* |
| **Type of Study** | *RCT* | | | |  |  |
|  | *Observational* | *Prospective* | | |  |  |
|  |  | *Retrospective* | | |  |  |
| **Participants** | - Adult patients (≥ 18 years of age) - Studies will remain eligible for inclusion if data on adolescent patients aged ≥ 13 years of age are included within the same cohort as adult patients. - Admitted with abdominal solid organ injury (liver, spleen, kidney) following blunt abdominal trauma - Initially treated nonoperatively | | | |  |  |
| **Types of Intervention** | - Early (< 48 hours from hospital admission) LMWH or UFH pharmacologic VTE prophylaxis initiation | | | |  |  |
| **Types of Comparators** | - Late ( ≥ 48 hours from hospital admission) LMWH or UFH pharmacologic VTE prophylaxis initiation | | | |  |  |
| **Types of Outcome Measures** | - Failure of nonoperative management   - Surgical intervention   - Angioembolization - Bleeding complications - pRBC transfusion requirements - VTE complications - Mortality | | | |  |  |
| *Include* | | | *Exclude* | | | |
| **Reasons for Exclusion:** | | | | | | |
| **Notes:** | | | | | | |
