# Supplementary figures and images for: Timing of pharmacologic venous thromboembolism prophylaxis initiation for trauma patients with nonoperatively managed blunt abdominal solid organ injury: a systematic review and meta-analysis
Source: World J Emerg Surg. 2022 Apr 25;17:19. doi: 10.1186/s13017-022-00423-1 (PMC9036793; doi:10.1186/s13017-022-00423-1)

**Appendix C.** Excluded full text citations and reasons.

**
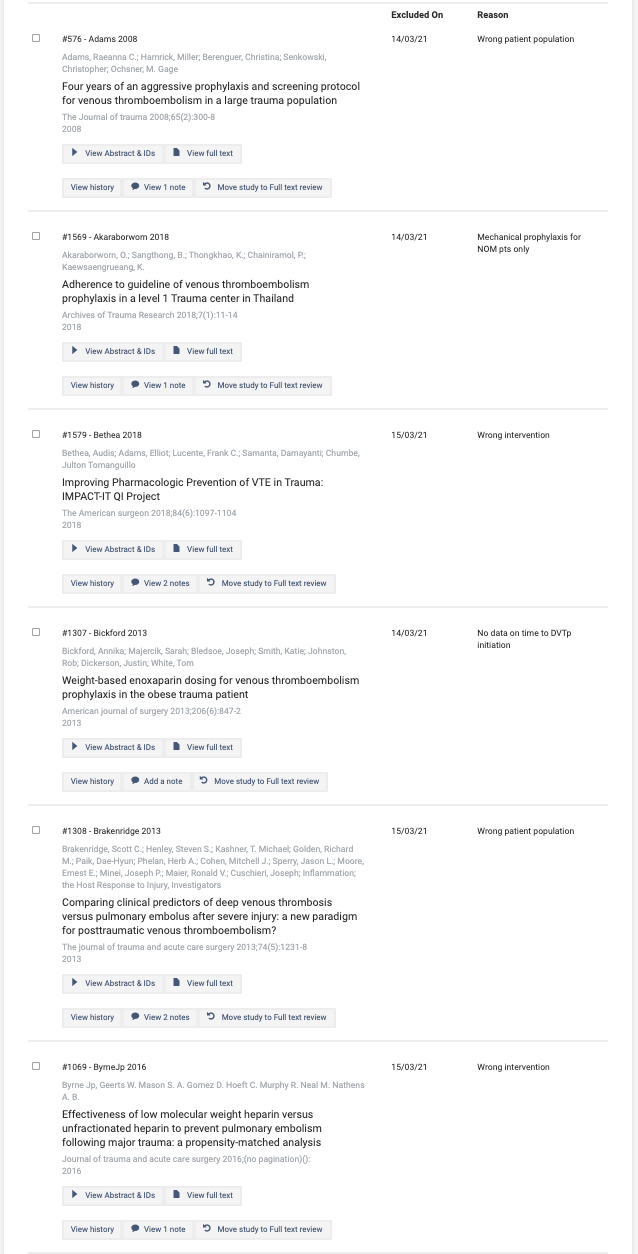
**

**
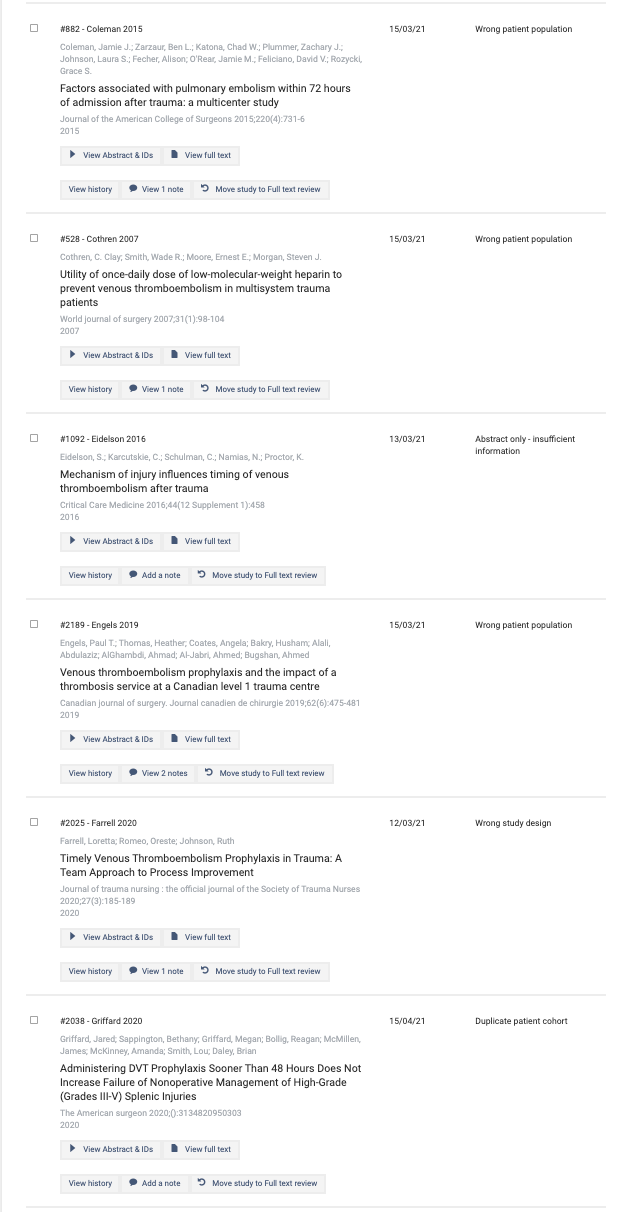

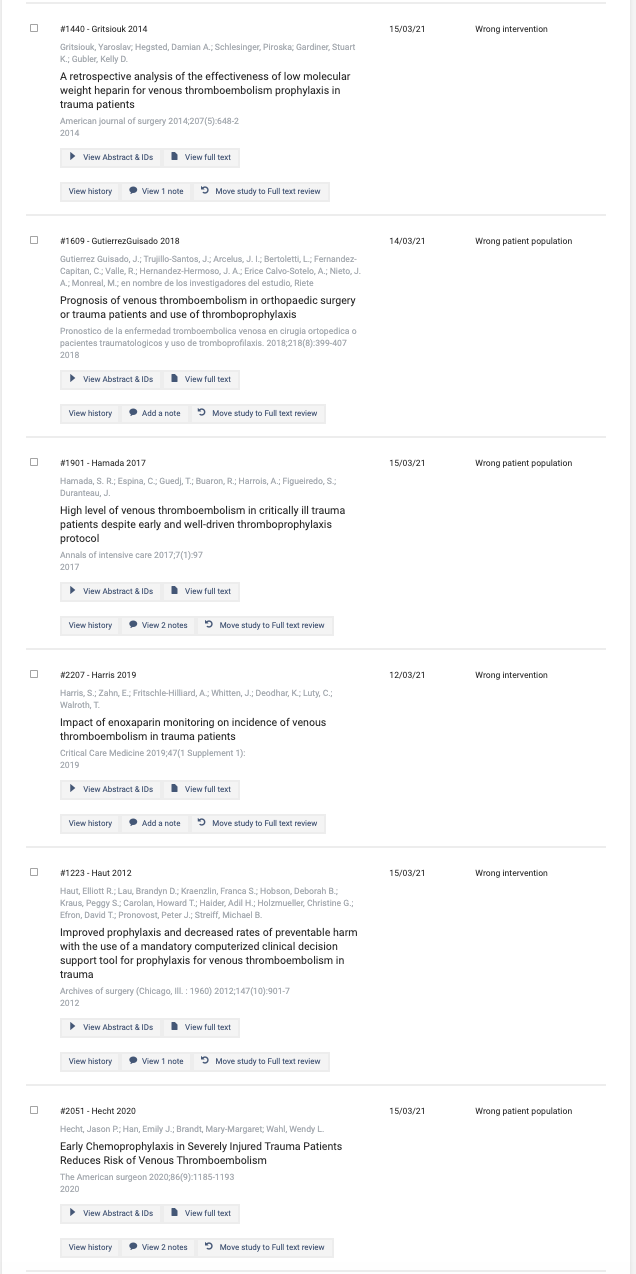

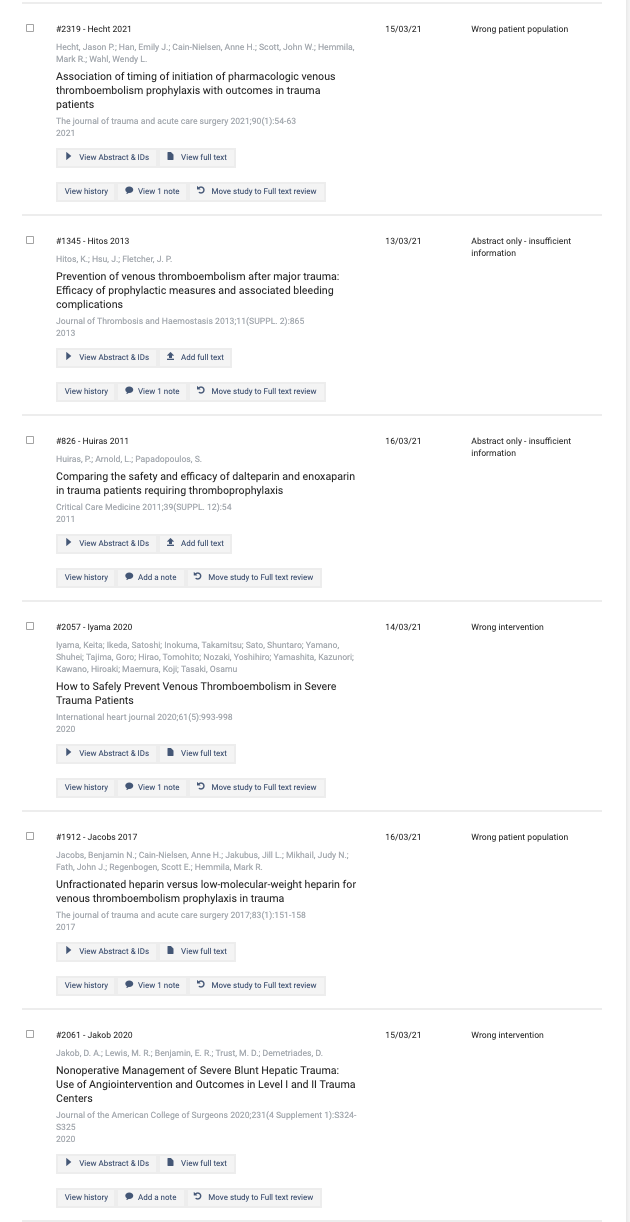

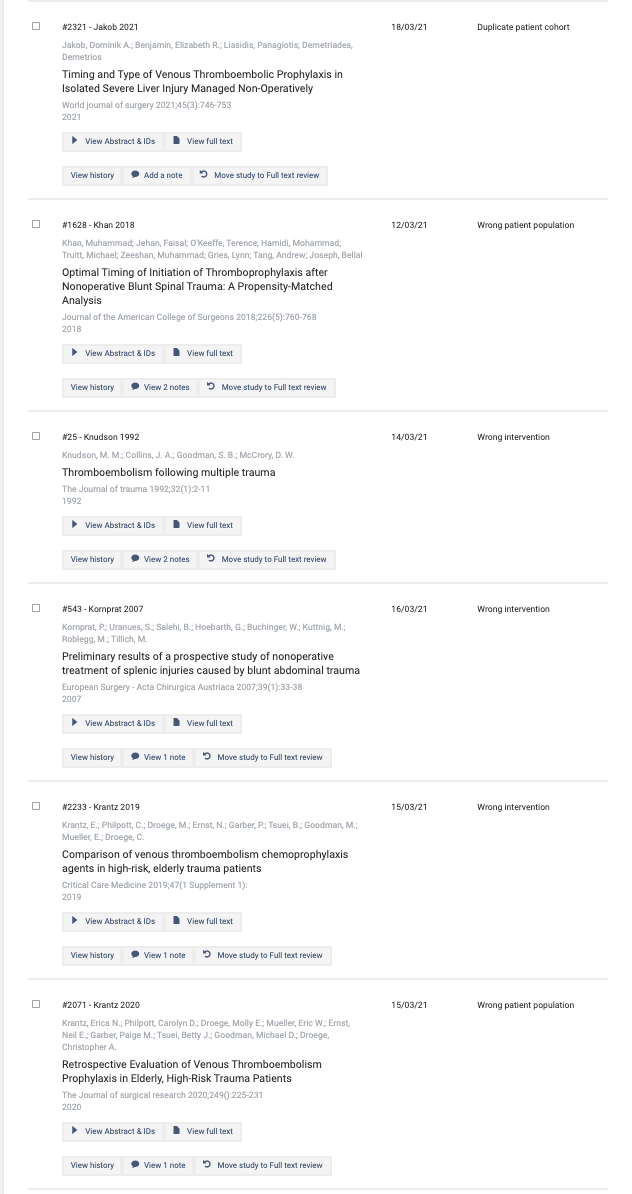

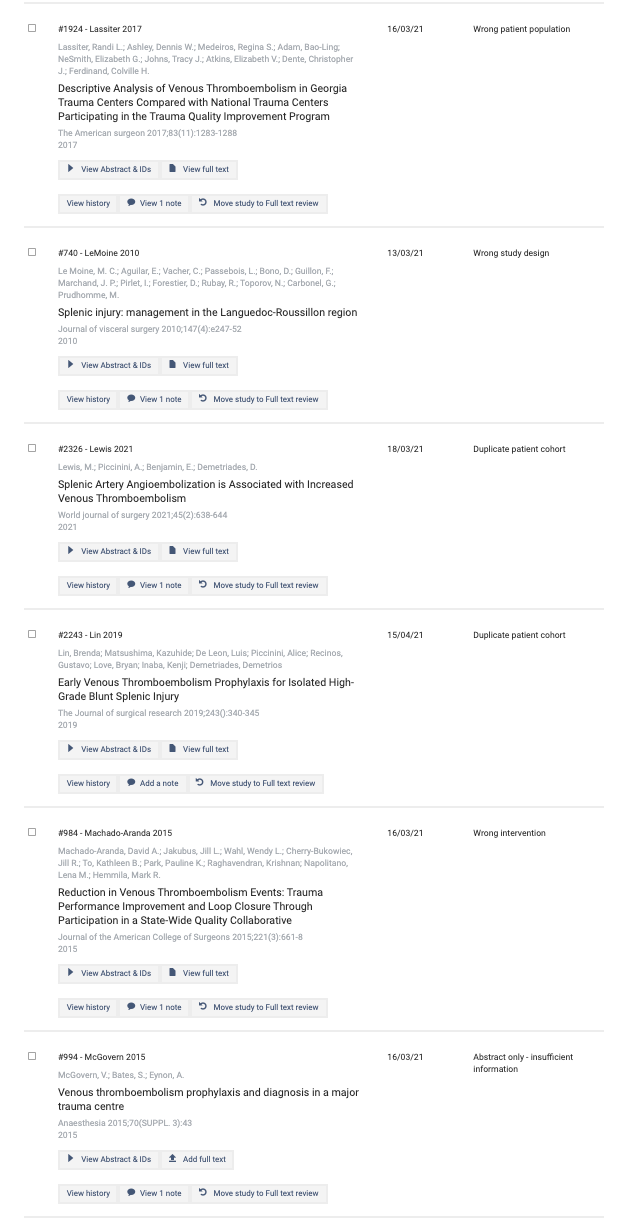

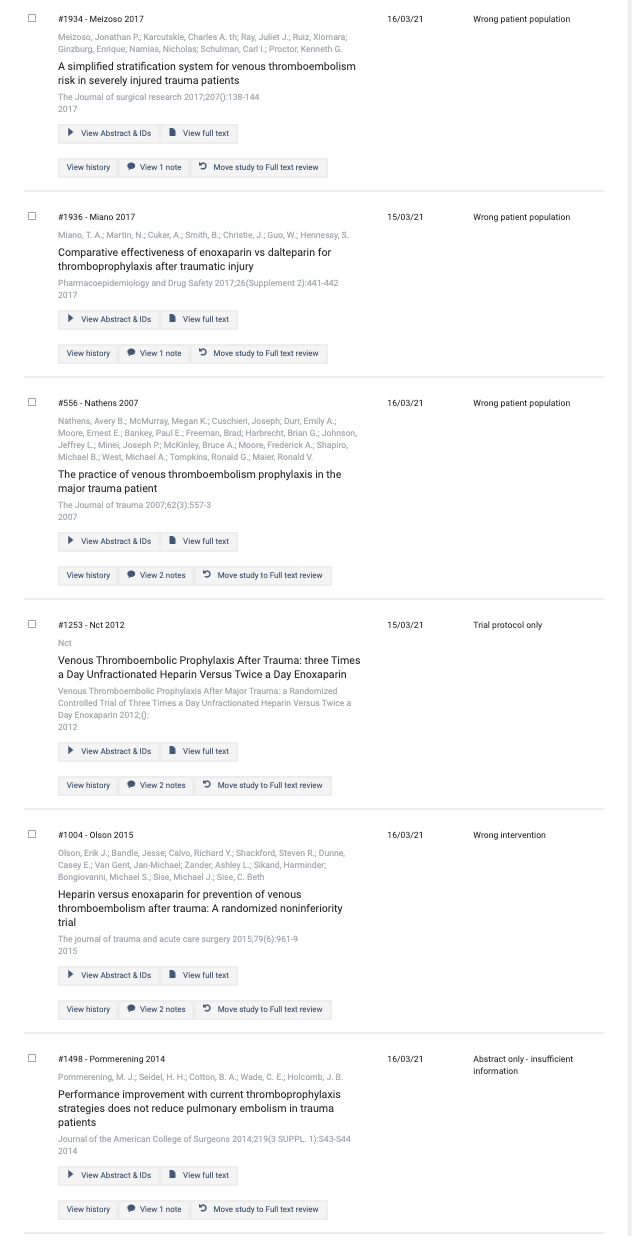

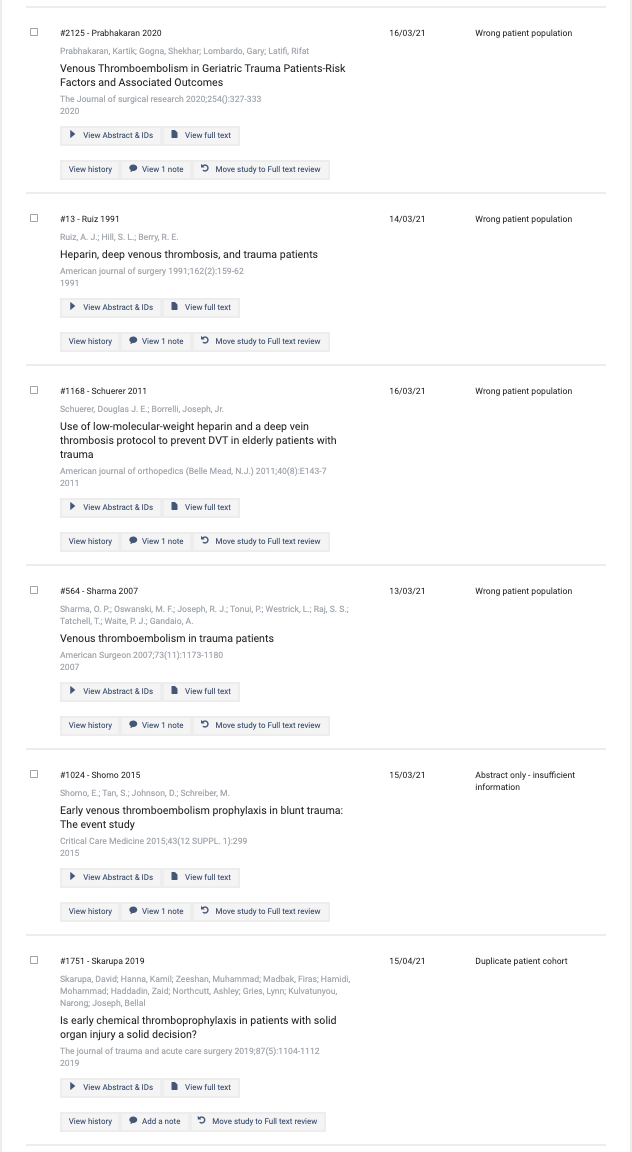

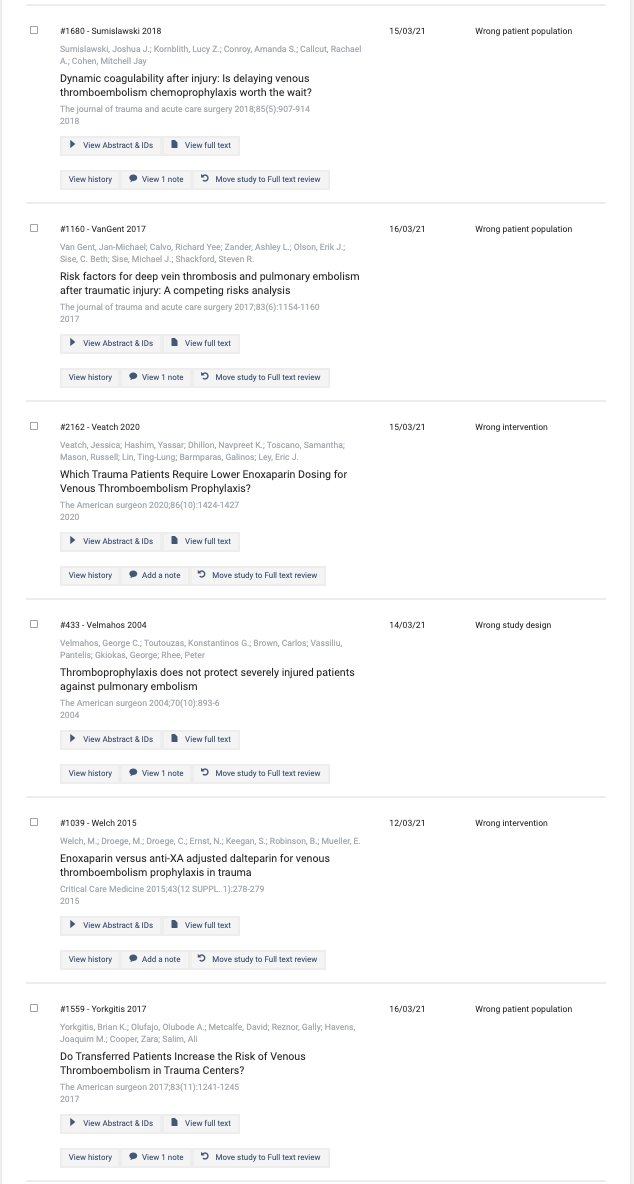
**

Supplement: Supplementary file 3 — Additional file 3: Appendix C. Excluded full text citations and reasons. [file 13017_2022_423_MOESM3_ESM.docx]

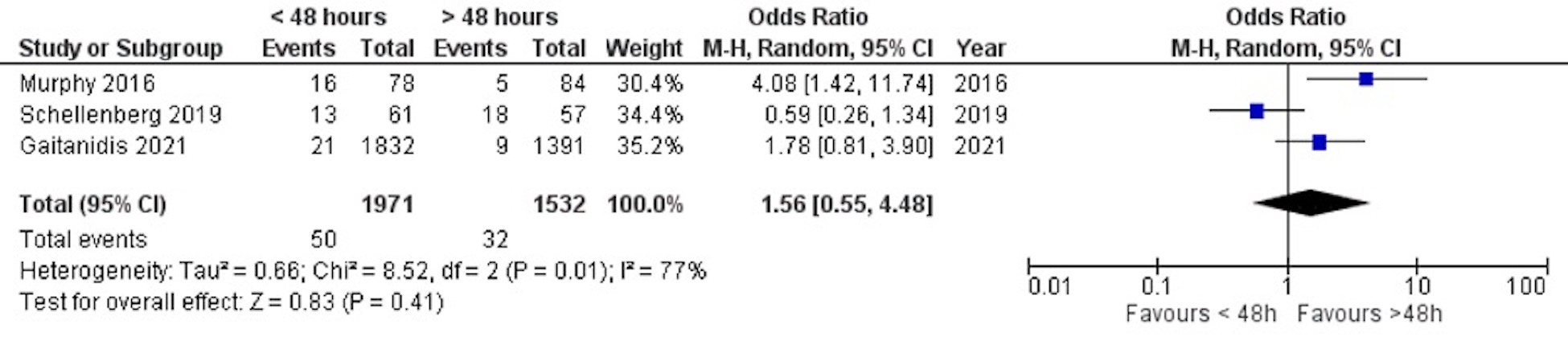

Supplement: Supplementary file 6 — Additional file 6: Fig. S1. Risk of transfusion after initiation of VTE prophylaxis (unadjusted data). [file 13017_2022_423_MOESM6_ESM.jpg]

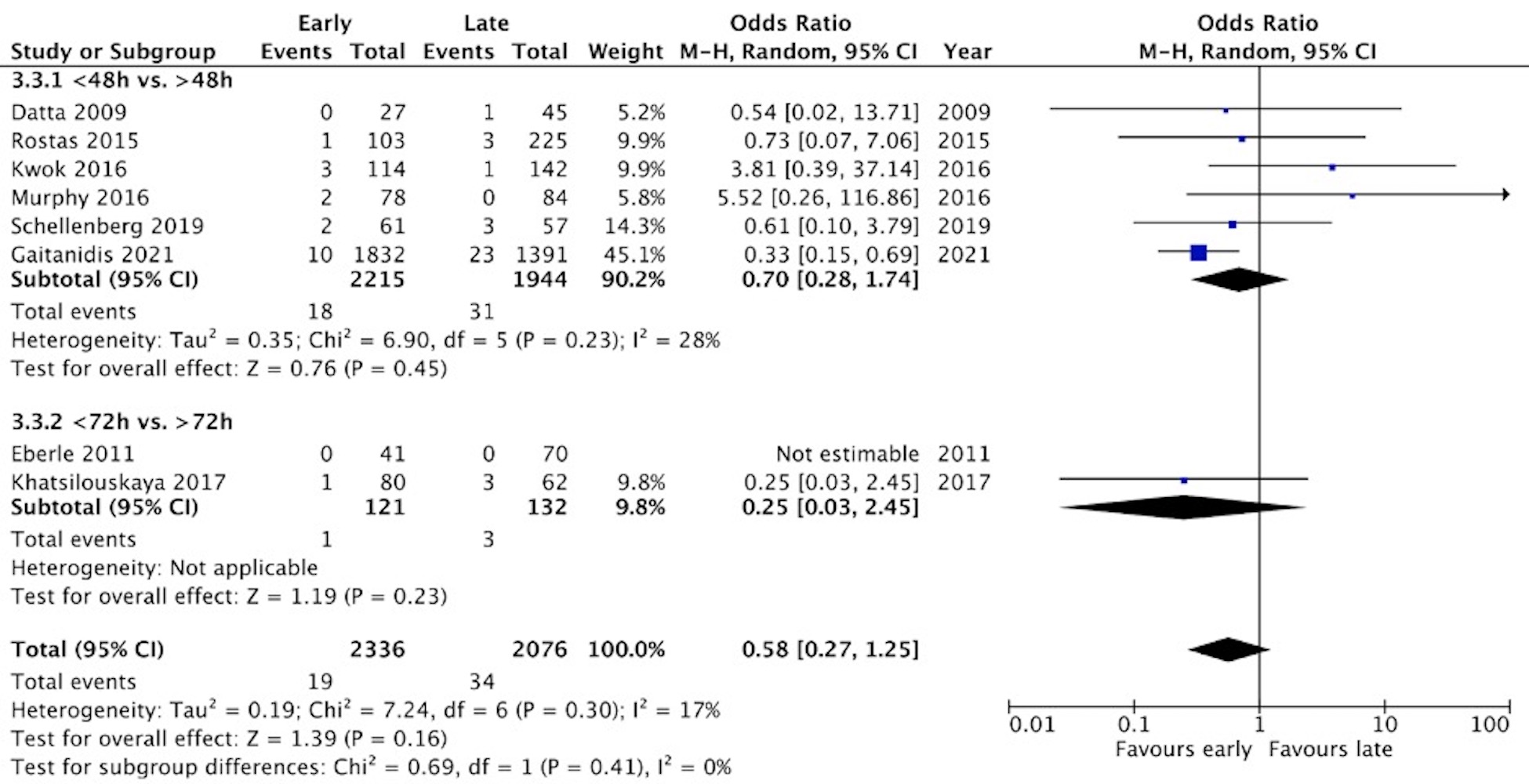

Supplement: Supplementary file 7 — Additional file 7: Fig. S2. Risk of PE (unadjusted data). [file 13017_2022_423_MOESM7_ESM.jpg]

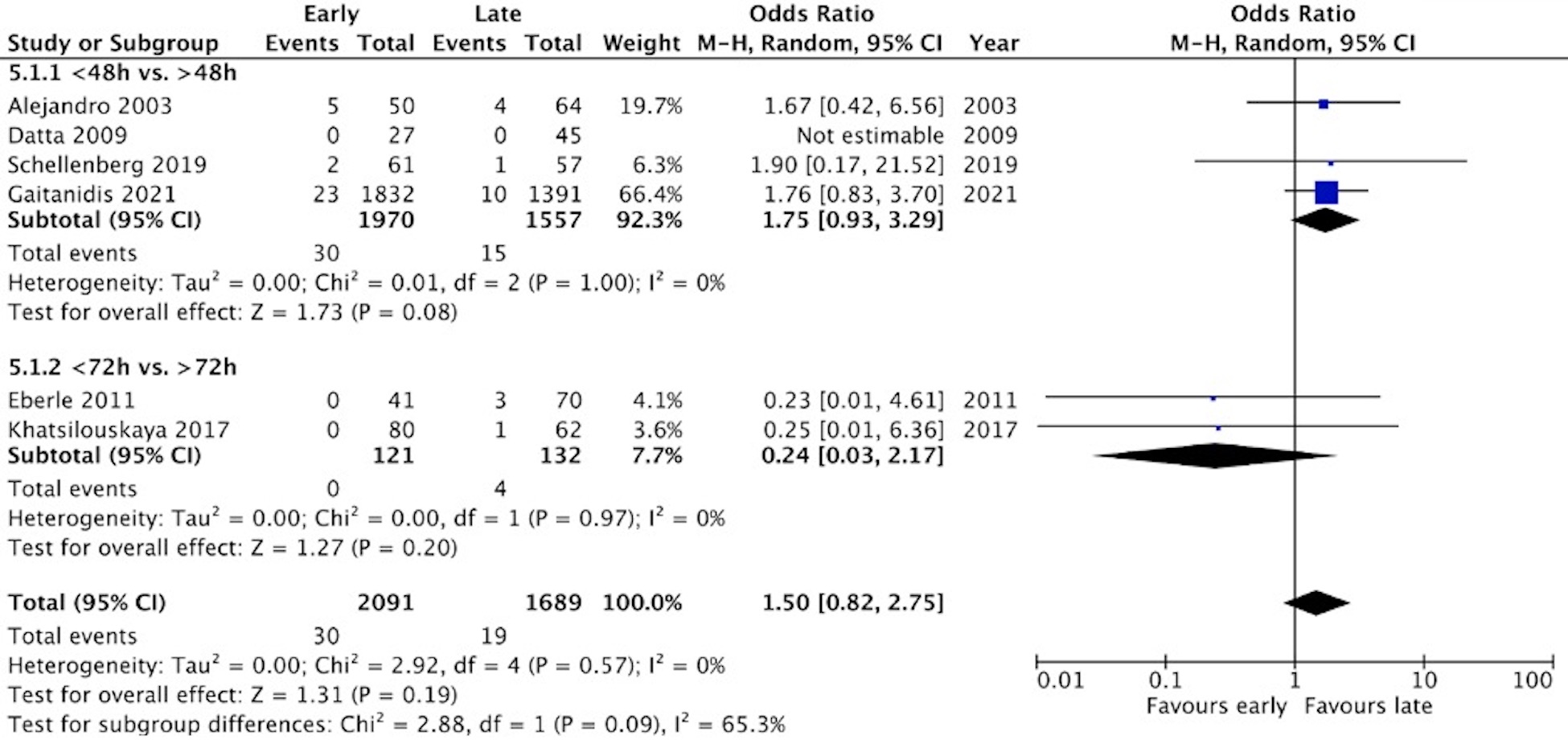

Supplement: Supplementary file 8 — Additional file 8: Fig. S3. Risk of mortality (unadjusted data). [file 13017_2022_423_MOESM8_ESM.jpg]
