# Supplementary material for: Timing of pharmacologic venous thromboembolism prophylaxis initiation for trauma patients with nonoperatively managed blunt abdominal solid organ injury: a systematic review and meta-analysis
Source: World J Emerg Surg. 2022 Apr 25;17:19. doi: 10.1186/s13017-022-00423-1 (PMC9036793; doi:10.1186/s13017-022-00423-1)
Supplement: Supplementary file 5 — Additional file 5: Table S1. AAST Grading of injuries in included studies. [file 13017_2022_423_MOESM5_ESM.docx]

**Supplementary Table 1.** AAST Grading of injuries in included studies.

| Study ID | Intervention Groups | Spleen | | | | | Liver | | | | | | Kidney | | | | | |
| --- | --- | --- | --- | --- | --- | --- | --- | --- | --- | --- | --- | --- | --- | --- | --- | --- | --- | --- |
|  |  | **Grade 1** (%) | **Grade 2** (%) | **Grade 3** (%) | **Grade 4** (%) | **Grade 5** (%) | **Grade 1** (%) | **Grade 2** (%) | **Grade 3** (%) | **Grade 4** (%) | **Grade 5** (%) | **Grade 6** (%) | **Grade 1** (%) | **Grade 2** (%) | | **Grade 3** (%) | **Grade 4** (%) | **Grade 5** (%) |
| Alejandro et al.  *(N=114)* | ≤ 48 hours | 76.0 | | 24.0 | | |  |  |  |  |  |  |  |  | |  |  |  |
|  | > 48 hours | 62.5 | | 37.5 | | |  |  |  |  |  |  |  |  | |  |  |  |
| Datta et al.  *(N = 72)* | ≤ 48 hours |  |  |  |  |  | 22.0 | 48.0 | 19.0 | 11.0 | 0 | 0 |  |  | |  |  |  |
|  | > 48 hours |  |  |  |  |  | 13.0 | 9.0 | 54.0 | 20.0 | 4.0 | 0 |  |  | |  |  |  |
| Eberle et al.  *(N=111)* | < 72 hours | 50 | | 50 | | | 44.4 | | 55.6 | | | | 50.0 | 50.0 | | | | |
|  | ≥ 72 hours | 60.0 | | 40.0 | | | 41.7 | | 58.3 | | | | 58.8 | 41.2 | | | | |
| Joseph et al.  *(N=116)* | ≤ 48 hours | 62.5 | | 37.5 | | | 53.5 | | 46.7 | | | | 55.6 | 44.4 | | | | |
|  | 48-72 hours | 45.5 | | 54.5 | | | 50.0 | | 50.0 | | | | 50.0 | 50.0 | | | | |
|  | ≥ 72 hours | 46.2 | | 53.8 | | | 28.6 | | 71.4 | | | | 37.5 | 62.5 | | | | |
| Rostas et al.  *(N=328)* | < 48 hours | 52.7 | 30.9 | 12.7 | 3.6 | 0 | 45.6 | 42.1 | 12.3 | 0 | 0 | 0 |  |  | |  |  |  |
|  | 48-72 hours | 48.4 | 29.0 | 16.1 | 6.5 | 0 | 33.3 | 30.6 | 22.2 | 13.9 | 0 | 0 |  |  | |  |  |  |
|  | > 72 hours | 36.7 | 36.7 | 19.4 | 7.1 | 0 | 35.8 | 36.6 | 19.5 | 8.1 | 0 | 0 |  |  | |  |  |  |
| Kwok et al.  *(N=256)* | < 24 hours | 35.0 | 52.0 | 13.0 | 0 | 0 |  |  |  |  |  |  |  |  | |  |  |  |
|  | 24-48 hours | 32.0 | 32.0 | 24.0 | 12.0 | 0 |  |  |  |  |  |  |  |  | |  |  |  |
|  | 48-72 hours | 28.0 | 38.0 | 14.0 | 11.0 | 9.2 |  |  |  |  |  |  |  |  | |  |  |  |
|  | > 72 hours | 32.0 | 35.0 | 25.0 | 55.0 | 3.0 |  |  |  |  |  |  |  |  | |  |  |  |
| Murphy et al.  *(N=162)* | < 48 hours | 74.0 | | 26.0 | | | 65.0 | | 35.0 | | | | 89.0 | 11.0 | | | | |
|  | ≥ 48 hours | 51.0 | | 49.0 | | | 68.0 | | 32.0 | | | | 62.0 | 38.0 | | | | |
| Khatsilouskaya et al.  *(N=142)* | ≤ 72 hours | 81.0 | | | 19.0 | | 77.0 | | | 35.0 | | | 72.0 | | 28.0 | | | |
|  | > 72 hours | 65.0 | | | 35.0 | | 49.0 | | | 51.0 | | | 55.0 | | 45.5 | | | |
| Schellenberg et al.  *(N=118)* | ≤ 48 hours | 9.0 | 55.0 | 32.0 | 0 | 5.0 | 23.0 | 42.0 | 19.0 | 16.0 | 0 | 0 | 24.0 | 29.0 | | 24.0 | 24.0 | 0 |
|  | > 48 hours | 24.0 | 48.0 | 19.0 | 9.0 | 0 | 15.0 | 38.0 | 31.0 | 0 | 15.0 | 0 | 18.0 | 6.0 | | 53.0 | 18.0 | 6.0 |
| Gaitanidis et al.  *(N=3,223)* | < 48 hours |  |  |  |  |  |  |  |  |  |  |  |  |  | |  |  |  |
|  | 48-72 hours |  |  |  |  |  |  |  |  |  |  |  |  |  | |  |  |  |
|  | > 72 hours |  |  |  |  |  |  |  |  |  |  |  |  |  | |  |  |  |
